# Treatment of Palm Oil Mill Effluent by a Microbial Consortium Developed from Compost Soils

**DOI:** 10.1155/2014/762070

**Published:** 2014-10-29

**Authors:** Charles O. Nwuche, Hideki Aoyagi, James C. Ogbonna

**Affiliations:** ^1^Department of Microbiology, University of Nigeria, Nsukka 410001, Nigeria; ^2^Department of Bioscience and Bioengineering, Graduate School of Life and Environmental Sciences, University of Tsukuba, Tennodai 1-1-1, Tsukuba-shi, Ibaraki 305-8572, Japan

## Abstract

A method for the aerobic treatment of palm oil mill effluent (POME) was investigated in shake-flask experiments using a consortium developed from POME compost. POME was initially centrifuged at 4,000 g for 15 min and the supernatant was enriched with (NH_4_)_2_SO_4_ (0.5%) and yeast extract (0.25%) to boost its nitrogen content. At optimum pH (pH 4) and temperature (40°C) conditions, the chemical oxygen demand (COD) of the effluent decreased from 10,350 to 1,000 mg/L (90.3%) after 7 days. The total bacterial population determined by plate count enumeration was 2.4 × 10^6^ CFU/mL, while the fungal count was 1.8 × 10^3^ colonies/mL. Bacteria of the genera *Pseudomonas, Flavobacterium, Micrococcus*, and *Bacillus* were isolated, while the fungal genera included *Aspergillus, Penicillium, Trichoderma*, and *Mucor*. When the isolated species were each inoculated into separate batches of the raw effluent, both pH and COD were unchanged. However, at 75 and 50% POME dilutions, the COD dropped by 52 and 44%, respectively, while the pH increased from 4 to 7.53. POME treatment by aerobic method is sustainable and holds promising prospects for cushioning the environment from the problems associated with the use of anaerobic systems.

## 1. Introduction

Palm oil mill effluent (POME) is an acidic, viscous, and voluminous colloidal suspension formed during palm oil processing from the mixed stream of sterilizer condensate, separator sludge, and hydrocyclone wastewater [1]. Depending on the method of processing, it is composed of 95-96% water, 0.6-0.7% oil, and 4-5% total solids [2]. POME is a highly polluting wastewater and causes considerable deterioration of soil and water quality when discharged untreated into the environment [3]. Recently, the enforcement of stringent laws pertaining to waste effluent discharges in some palm oil producing communities in Nigeria have challenged researchers to investigate new approaches for the effective management of POME.

Several physicochemical techniques such as adsorption [4], solvent extraction [2], chemical-biological sedimentation [5], coagulation-flocculation [6], and membrane technology [7] have been applied in mitigating the polluting effects of POME but the reported outcomes have not produced acceptable results. Biological treatment methods, especially ponding, are the most common treatment system implemented perhaps due to their low capital cost [8] but the effectiveness of these methods is plagued by several draw-backs such as long retention time and start-up periods, necessity for large digesters, sensitivity of microorganisms to variations in environmental conditions, and the emission of corrosive and odorous biogas [9].

Currently, microorganisms used for the treatment of POME and other oil processing wastes have mainly been single species [10, 11]. However, due to differences in the characteristics of most effluents, it could be difficult for one organism to metabolize all the polluting components to acceptable discharge characteristic [12]. Microbial monocultures metabolise only a limited range of substrates but a mixed microbial community may be more efficient for treatment or remediation due to their broad enzymatic capacities [13]. In the anaerobic (ponding) method of POME treatment for instance, different populations of microorganisms are mobilized in a succession of distinct biochemical phases to bring about higher COD removal than any of the individual component species could achieve [14]. The use of mixed cultures in biodegradation studies has numerous advantages which include higher tolerance to perturbations such as changes in nutrient, pH, temperature, and pollutant concentrations [15].

The practice of diluting effluents with water prior to treatment is common but it increases the effluent volume several folds making it not only challenging and expensive to handle but significantly heightens the risk of environmental damage through seepage into water and soil microhabitats. Presently, modern treatment methods require some form of concentration as a prestep before appropriate treatment strategy is implemented. Although the use of a single organism in the aerobic treatment of POME has been reported [10], there still exists paucity of scientific information on the application of mixed cultures. In the present study, therefore, a method for the treatment of POME using a microbial consortium developed from compost soil is presented.

## 2. Materials and Methods

### 2.1. Palm Oil Mill Effluent (POME)

POME used in the present study was obtained fresh from a local palm oil processing mill at Nsukka, Enugu State, Nigeria. It was shaken in a 1 L measuring cylinder for 30 s and then kept for 1 h to allow for sedimentation of solids. Subsequently, the effluent was centrifuged at 4,000 g for 15 min for total elimination of solids. The resulting supernatant was distributed into 500 mL Erlenmeyer flasks. Prior to inoculation, each 100 mL volume was supplemented with 0.5% (NH_4_)_2_SO_4_ and 0.25% yeast extract. The medium was sterilized at 121°C for 15 min at 15 psi.

### 2.2. Isolation and Identification of Microorganisms

For the enumeration of the microbial populations, serial tenfold dilutions were made on supernatants from the compost suspension. Aliquots of 100 *μ*L were taken from both low and high dilutions and spread inoculated over the surfaces of replicate sterile plates of nutrient agar and potato dextrose agar (PDA) for the growth of bacterial and fungal colonies, respectively. After 48 h at 37°C, bacterial colonies were counted while the fungal cultures were kept at 30°C for 4-5 days. Emerging discrete colonies were streaked onto new agar plates in order to isolate each strain in pure culture. Genotypic identification was done based on Bergey's Manual of Systemic Bacteriology. For the fungal isolates, micromorphological features were compared against the genus descriptions of Hoog et al. [16].

### 2.3. Nitrogen Sources

Various nitrogen containing inorganic and organic compounds were examined for their effect on the rate of COD reduction by the inoculated cultures. The method employed was to substitute (NH_4_)_2_SO_4_ for KNO_3_, NH_4_NO_3_, CO(NH_2_)_2_, NH_4_Cl, peptone, or yeast extract in the medium while maintaining other factors constant. For the purpose of this experiment, the usual yeast extract supplementation was excluded.

### 2.4. Effect of Individual Species on COD Reduction

The isolated organisms were inoculated into separate batches of POME to examine their impact on the COD of the effluent. Bacterial cultures were propagated in freshly prepared nutrient broth (Lab M) by inoculation of 1 loopful of each strain into 100 mL medium. Incubation was carried out at 30°C to a final cell concentration of 10^7^ CFU/mL. As previously indicated, the medium was centrifuged, and the pellets were washed twice in sterile saline and resuspended in a 5 mL volume before inoculating the POME medium. The fungal spores were transferred aseptically onto sterile surfaces of freshly prepared PDA plates and incubated for 5 days. Thereafter, the surfaces of the plates were flooded with 5 mL of distilled water and scrapped gently by a sterilized inoculating needle to dislodge the fungal spores from the mycelia fragments. The suspension was transferred to a clean receptacle. The pooled spore suspension was centrifuged at 4,000 g for 10 min, washed, and resuspended in another 5 mL of distilled water. The concentrated spore suspension was then used to inoculate a POME medium after adjusting the spore density to 1.0 × 10^7^ spores/mL with the aid of a Neubauer haemocytometer. Fermentation was conducted for a total of 7 days as previously indicated.

### 2.5. Development of the Mixed Culture Inoculum

Materials from POME composts (dump sites) were collected from ten random locations at 5–15 cm depth using a 2.5 cm soil auger. The samples were carried inside sterile labeled polyethylene plastic bags protected from direct sunlight and transported to the laboratory for further processing. On arrival, 5 g of the homogenized sample was transferred to a 250 mL Erlenmeyer flask containing 45 mL of sterile physiological saline (PS; Oxoid). The flask was agitated in a shaker at 100 rpm for 2 h at 26 ± 2°C to dissolve clumps and disperse organic materials. The solid suspension was centrifuged at 1,500 g for 15 min and the supernatant (10%) was inoculated into a previously sterilized and cooled mineral salts medium (MSM) composed of (g/L) (NH_4_)_2_SO_4_(5), Na_2_PO_4_(6), KH_2_PO_4_(2), MgSO_4_(3), CaCl_2_·2H_2_O (3), and POME 10 mL as the sole carbon source. The pH of the medium was adjusted to 4 in line with the pH of the soil prior to sampling. The medium was enriched through weekly transfers of 10% (v/v) of the culture supernatant to a fresh medium over a total of six weeks.

### 2.6. Microorganisms and Culture Conditions

Flasks in the last acclimation stage were withdrawn and the content of one Erlenmeyer flask was centrifuged at 10,000 g for 15 min. The microbial pellet formed was washed in normal sterile physiological saline (0.85% NaCl) before resuspending in another 5 mL solution to form the microbial inoculum. The content of the test tube was then emptied into a 500 mL Erlenmeyer flask containing 95 mL of the POME. Fermentation was conducted at 100 rpm, pH 4 at 30°C for 7 days except otherwise indicated. Samples taken periodically were passed through Millex 0.22 *μ*m filters (Millipore SA, France) to eliminate microbial cells and mycelia before analyzing for COD and pH.

### 2.7. Optimization of pH and Temperature Conditions for Consortium Inoculum

The medium was adjusted before sterilization to pH values of  3, 4, 5, 6, or 7 using 1 M H_2_SO_4_ and 1 M NaOH. The cultures were inoculated and incubated at 30°C for 7 days. Later, media adjusted to the best pH condition were treated as before and incubated at temperatures of 30, 35, 40, 45, or 50°C for 7 days. The cell free filtrates withdrawn periodically were evaluated for changes in COD and pH.

### 2.8. Analytical Methods

The pH, oil and grease (O and G), total solids (TS), and total suspended solids (TSS) were determined by standard methods [17]. Total soluble carbohydrate (TSC) was analyzed by the phenol-sulphuric acid method [18]. Chemical oxygen demand (COD), ammonia-nitrogen (NH_3_-N), total nitrogen (N), and nitrates (NO_3_^−^) were measured by the Hach Spectrophotometric method (DR/4000, Hach Co., Ltd., Tokyo). Phenol was estimated by the phenol test kit (Wako Pure Chemical Ind., Osaka).

### 2.9. Statistical Analysis

The presented results are the means of triplicate determinations ± standard deviation. Where applicable, the completely randomized one-way analysis of variance was used to determine the level of significance of the treatments. The statistical software IBM-SPSS (2008 version) was used in the calculations.

## 3. Results

### 3.1. Characteristics of POME Used in the Study

The major characteristics of POME used in the present study are summarized in Table 1. It had an initial pH of 3.98 ± 0.02 and oil and grease concentration of 2,800 ± 300 mg/L. The COD and TSC were 60,400 ± 784 and 4470 ± 230 mg/L, respectively. TS was 27,300 ± 640 mg/L, while TSS was 24,000 ± 690 mg/L. Phenol was detected at the concentration of 100 ± 00 mg/L. Among the nitrogen containing compounds, ammonia (nitrogen) had a concentration of 410 ± 20 mg/L, while the total nitrogen was 1,850 ± 50 mg/L. The nitrate concentration was found to be 500 ± 20 mg/L. When the supernatant of the POME was analyzed after sedimentation, pH was 3.97 ± 0.03 but O and G level reduced to 840 ± 40 mg/L. COD decreased to about 8,700 ± 500 mg/L, while the TSC concentration dropped to 580 ± 65 mg/L. Both TS and TSS were nondetectable. Phenol concentration was constant (100 ± 00 mg/L) but ammonia nitrogen reduced to 110 ± 07 mg/L. The concentration of both nitrogen (total) and nitrates dropped to 90 ± 04 and 40 ± 05 mg/L, respectively. Analysis of the final effluent supplemented with both (NH_4_)_2_SO_4_ and yeast extract indicates that the pH increased to 4.04 ± 0.05, while O and G did not change (860 ± 25 mg/L). COD rose to 10,350 ± 430 mg/L, while the TSC increased marginally to 660 ± 30 mg/L. Both TS and TSS were not detected but phenol concentration remained 100 ± 00 mg/L. Nitrogen concentrations were significantly affected by the addition of nitrogen sources. In particular, nitrogen (ammonia) and total nitrogen concentrations were 1,250 ± 85 and 2,835 ± 130 mg/L, respectively, while the concentration of nitrates was 840 ± 30 mg/L. When the supplemented POME was inoculated with both single strain and mixed cultures in separate batches, significant changes were found when the final effluents were analyzed. Final pH was 4.03 ± 0.02 in the single strain treatment but in the mixed cultures, it was 7.79 ± 0.15. The concentration of O and G in the former was 530 ± 20 mg/L but completely eliminated in the latter. COD content of the single strain treatment was 10,360 ± 350 mg/L. This value decreased to 1,000 ± 100 mg/L (90.3%) in treatment containing the mixed cultures. One other interesting discovery in this batch was the complete disappearance of phenol even though its initial concentration of 100 mg/L persisted in the batch treated with a single strain. Results also indicate that the treatments affected the nitrogen content of the effluents significantly (*P* < 0.05). In the single strain treatment, ammonia (nitrogen) concentration was 1,520 ± 55 mg/L but this decreased to 780 mg/L in the mixed culture treatment. There was no significant change in the total nitrogen content of the former (single strain), but in the latter, total nitrogen dropped to 1,920 ± 30 mg/L. Nitrate concentrations in both effluents were 830 ± 20 mg/L and 750 ± 30 mg/L, respectively.

### 3.2. Enumeration of the Microbial Populations and Identification of the Adapted Species

Total plate count estimation of the microbial load of the compost samples showed that the bacteria population was 2.4 × 10^6^ CFU/mL, while fungi were 1.8 × 10^3^ colonies/mL (Table 2). However, biochemical identification of the organisms isolated after the adaptation experiments reveals that the bacterial community was composed of four main genera, namely,* Pseudomonas, Flavobacterium, Micrococcus*, and* Bacillus*. The species of the* Pseudomonas* and* Bacillus* genera isolated were* Pseudomonas fluorescens* and* Bacillus subtilis*. Among the isolated fungal genera were* Aspergillus, Penicillium, Trichoderma*, and* Mucor*.* Aspergillus niger* was the most frequently isolated fungus, while* Trichoderma* was the least.

### 3.3. Effect of Different Nitrogen Compounds on COD Reduction

**Figure 1:**
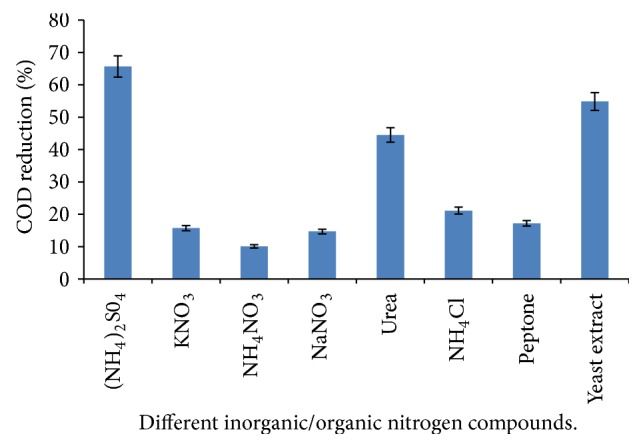
Effect of different nitrogen compounds on COD reduction.

Among the range of both organic and inorganic compounds tested (Figure 1), (NH_4_)_2_SO_4_ promoted the highest COD removal (65.6%). Subsequently it was selected as a nitrogen supplement for the medium. Yeast extract and urea had 55% and 44.5% removal efficiencies, respectively. NH_4_NO_3_ elicited the least COD decrease (10%) while with KNO_3_, NaNO_3_, and peptone, less than 20% COD decrease was achieved.

### 3.4. Effect of Individual Cultures on COD Reduction

**Figure 2:**
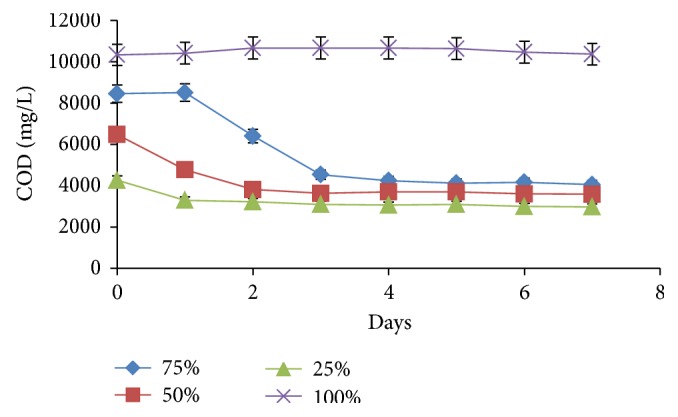
Effect of POME concentration on COD changes during treatment by an isolate (*Aspergillus* sp.).

**Figure 3:**
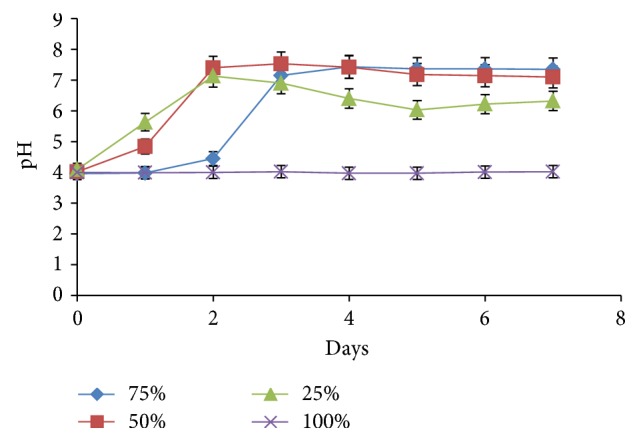
Effect of POME concentration on pH changes during treatment by an isolate (*Aspergillus* sp.).

When individual cultures were inoculated into separate batches of the raw POME media, results indicated that the organisms did not achieve significant reduction in the COD of the effluent (Figure 2). COD decreased between 0 and 0.65% in media inoculated with both bacterial and fungal strains (data not shown). When different dilutions of the medium were used, significant changes in COD took place. At 75% POME concentration, COD dropped by 52% (8,455 to 4,055 mg/L) while in the 50 and 25% POME, COD losses were 44% (6,475 to 3,595 mg/L) and 30% (4,265 to 2,975 mg/L), respectively. As shown in Figure 3, pH was unchanged (pH 4) in the 100% POME but at higher dilutions, pH went slightly beyond the neutral mark. At 75% concentration, pH rose after a 36 h lag to a peak value of 7.43. At other concentrations, pH values of up to 7.53 were achieved in the 50% POME while in the 25% concentration, pH reached 7.13.

### 3.5. Effect of pH and Temperature Changes on COD Kinetics

**Figure 4:**
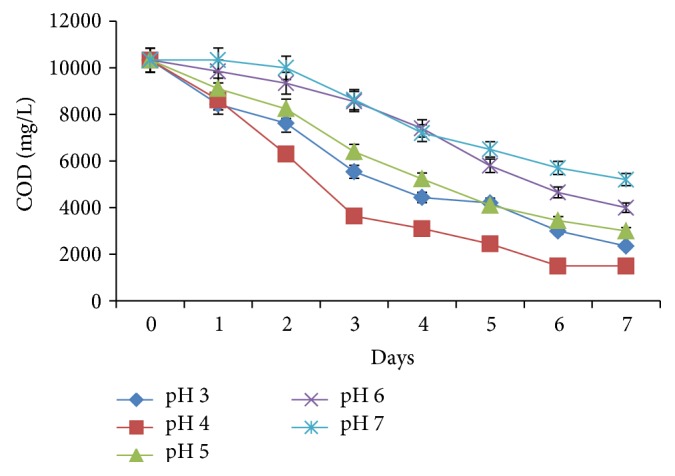
Effect of pH on COD of the effluent during treatment.

As fermentation progressed to 3rd day, COD in the effluents of pH 4 dropped to 3,640 mg/L (64%) while in the samples adjusted to pH 3, 46% (5,540 mg/L) decrease was achieved (Figure 4). By the 6th day, COD had reduced to 1,500 mg/L at pH 4, representing 85.5% reduction. On the other hand, at pH 3, the COD decreased steadily up to the 7th day with a total loss of 77% (7,985 mg/L). COD kinetics at both pH 5 and 6 showed that COD dropped to 9,100 mg/L (12%) but at pH 6, only 4.47% reduction was achieved. By the 3rd day, nearly 40% (3,925 mg/L) of the COD had been removed at pH 5 while only 17% (1,757 mg/L) was lost at pH 6. Total COD losses at both pH (5 and 6) conditions were 71 and 61%, respectively. At pH 7, COD was unchanged after 48 h but by the 3rd day, 16.5% (1,705 mg/L) of the COD had been removed. By the 7th day, approximately 50% (5,200 mg/L) of the organic load had been removed.

**Figure 5:**
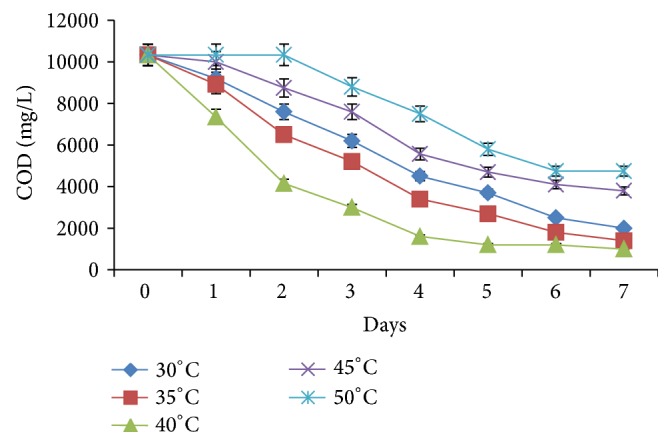
Effect of temperature on COD of the effluent during treatment.

Temperature also had significant effect on the COD changes in the medium (Figure 5). At 30 and 35°C, the total COD removal was 80.6% (8,335 mg/L) and 86.5% (8,935 mg/L), respectively. At 40°C, the COD dropped rapidly to 4,150 mg/L (60%) within 2 days and further to 1,600 mg/L (84.5%) by the 4th day and finally to 1,000 mg/L (90.3%) by the 5th day. Afterwards, COD was stable until the end of the treatments. The rates of COD reduction at 45 and 50°C were lower than those observed at lower temperatures. Initially, a lag appeared before measurable changes in COD were observed. At 45°C, the lag lasted for 24 h but at 50°C, it took longer than 48 h before cultures could become metabolically active. Despite the initial delays, total COD decrease at 45°C was 63% (6,535 mg/L), while at 50°C, 54% (5,585 mg/L) COD was lost.

## 4. Discussion

### 4.1. Characteristics of POME Used in the Study

The sedimentation process resulted in significant (*P* < 0.05) reduction in the major pollution indices of the wastewater (Table 1). O and G, for instance, decreased by 70% (2,800 to 840 mg/L). The COD and TSC dropped by 85.6% (60,400 to 8,700 mg/L) and 86.9% (4,470 to 580 mg/L), respectively, while both the TS and TSS were completely eliminated. The sediments or sludge is composed of lignocelluloses and other polysaccharides [19] and may be processed into biohydrogen [20], fertilizers [21], enzymes [22], and citric acid [23]. Nitrogen is present in POME in organic (protein) forms but with time, the organic nitrogen converts to ammonia [24]. Sedimentation also diminished the nitrogen content of the POME even further. However, supplementation with ammonium sulphate and yeast extract provided adequate supplies of the required compounds. In treatments containing separate batches of both single and mixed cultures, it was found that the mixed populations promoted higher COD removal than the single strain. The pH of the final effluent (7.79 ± 0.15) was also found to be within the acceptable discharge limit for treated effluents. Although significant concentration of O and G was found after treatment with the single strain (530 ± 20 mg/L), it was completely metabolized in the batch containing mixed cultures. The increase in the concentration of TSC (845 ± 24 mg/L) might result from the accumulation of glycerol following the enzymatic hydrolysis of oils in the wastes. Supplementation with ammonium sulphate and yeast extract boosted the nitrogen content of the effluent. However, after treatment with the mixed cultures, the compounds decreased substantially unlike the single strain treatment.

### 4.2. Enumeration of the Microbial Populations and Identification of the Adapted Species

A total of four bacterial and five fungal genera were isolated (Table 2). Their compositions and density were identical to previous report by Ugoji [25] on POME microflora. Of the four bacterial isolates,* B. subtilis* was the most frequently isolated (data not shown). The high temperature of the compost environment may have promoted the domination of the* Bacillus* strains over the other bacterial genera.* Bacillus* has been widely isolated during the thermophilic stages of composting [26]. In addition, the frequency of isolation of* P. fluorescens* and* B. subtilis* might be connected with the abundance of cellulose in the waste. Cellulose is dominant in plant materials found in POME composts and both organisms have been shown to be good cellulase producers [27]. Equally, the lipase producing property of* P. fluorescens* has been acknowledged [28]. Among the isolated fungi,* Trichoderma* sp. has been widely studied for cellulase production [29]. Members of the* Aspergillus* genera were found to be more prominent than the other moulds. The versatility of members of this group might explain their preponderance and domination in virtually any habitat [30].* Aspergillus, Penicillium*, and* Mucor* are among the most reported genera of lipase-producing filamentous fungi [31].

### 4.3. Effects of Different Nitrogen Compounds

Oil mill effluents do not contain sufficient nitrogen and phosphorus compounds for an effective aerobic treatment process [11]. Therefore, adequate supplementation has to be made in order to satisfy the nutritional requirement of the metabolizing microbiota. In the present study, (NH_4_)_2_SO_4_ was found to have promoted the highest rate of COD removal (65.6%) and was therefore selected. Several previous reports have highlighted the need for the addition of nitrogen compounds to boost the rate of remediation or treatment. Scioli and Vollaro [32] used 0.6% (NH_4_)_2_SO_4_ and 0.1% yeast extract in the treatment of olive oil effluent by* Yarrowia lipolytica*. In the absence or inadequate nitrogen supplementation, COD removal can be as low as 1.47% [11] and this may make the biological process unfeasible and uneconomical.

### 4.4. Effect of Individual Species on COD Kinetics

At lower POME concentrations, the organisms achieved significant (*P* < 0.05) reduction in the COD of the effluent but were unable to metabolize the full strength wastewater (Figures 2 and 3). The activities of the different strains were found to be limited, perhaps due to the presence of inhibitory components such as phenol, flavonoids, and alkaloids [33]. These compounds are known to be present in POME and have been reported to display both antibiotic and phytotoxic properties [34]. However, the mixed microbial populations were not negatively affected perhaps due to their wider enzymatic potentials and proven tolerance to high pollutant concentrations [15]. Dilution has been shown to reduce the impact of these compounds, enabling the microbes to thrive and metabolize the organic materials present in the effluent. Although dilution is seen as an option, it results in increase in the volume of the effluent creating a requirement for larger spaces and equipment and making the treatment efforts more laborious and capital intensive. Also, data obtained from treatment at diluted concentrations were statistically (*P* < 0.05) lower than treating the raw POME with a community of organisms. It is noteworthy though that the effects observed with respect to the use of the consortium are not due to the isolated species alone because less than 10% of all microorganisms are cultivable [35].

### 4.5. Optimization of pH and Temperature

In the present study, both pH and temperature conditions (Figures 4 and 5) were found to affect the efficiency of POME treatment in line with previous studies by Kwapisz et al. [36]. The pH 4 was found to be the optimum pH for COD reduction. This value is identical to most POME which is usually low due to accumulation of fatty acids [37]. Although pH 4 was the optimum pH value, COD decrease was substantial at both lower and higher pH conditions.

The variations in temperature equally resulted in a spectrum of COD changes. At 40°C, total COD reduction was the highest (92%) with value getting as low as 1,000 mg/L. At higher temperatures, COD reduction declines due to decrease in the metabolism of cultures as a result of denaturation of key proteins [38]. Although the decrease in COD dropped at 50°C to less than 54%, yet treatment was substantial even at such high temperature. This finding is noteworthy because since the system was operated by thermotolerant organisms, the requirement for cooling was found not to be necessary. Yu et al. [39] reported that operating a flow reactor at 55°C achieved higher substrate degradation rate, biogas production rate, and specific rate of aqueous product formation than when operated at 37°C.

## 5. Conclusion

The use of a microbial consortium in the aerobic treatment of POME was investigated in the present study. At optimum conditions, the organisms were found to be more beneficial for large-scale remediation process than any of the constituting individual species. The absence of the requirement for dilution coupled with the ability of the microbes to overcome the growth and metabolic challenges imposed by the presence of inhibitory components in the raw POME highlights the potential value of this method for the treatment of the effluent or remediation of environment polluted by the oil processing waste. Research efforts are presently ongoing to scale up the process from the shaker-flask experiments into pilot scale bioreactor.

## Figures and Tables

**Table 1:** Characteristics of the different POME treatments used in the study.

Parameters (mg/L)	Raw POME	POME supernatant	POME supernatant + (NH_4_)_2_SO_4_ (0.5%) + yeast extract (0.25%)	POME (100%) after treatment with single strain	POME after treatment with mixed cultures
pH^*^	3.98 ± 0.02	3.97 ± 0.03	4.03 ± 0.02	4.03 ± 0.02	7.79 ± 0.15
Oil/grease (O and G)	2,800 ± 300	840 ± 40	860 ± 25	530 ± 18	ND
COD	60,400 ± 784	8,700 ± 500	10,350 ± 430	10,360 ± 350	1,000 ± 100
Total soluble carbohydrate	4,470 ± 230	580 ± 65	660 ± 30	645 ± 25	845 ± 24
Total solids (TS)	27,300 ± 640	ND	ND	ND	ND
Total suspended solids (TSS)	24,000 ± 690	ND	ND	ND	ND
Phenol	100 ± 00	100 ± 00	100 ± 00	100 ± 00	ND
Ammonia (nitrogen)	410 ± 20	40 ± 05	1,250 ± 85	1,520 ± 55	780 ± 20
Nitrogen (total)	1,850 ± 50	110 ± 07	2,735 ± 130	2,650 ± 90	1,920 ± 75
Nitrates	500 ± 20	90 ± 04	840 ± 30	830 ± 20	750 ± 30

ND: not detected.

^*^Not measured in mg/L.

**Table 2:** Enumeration and identification of the adapted microbial populations in the samples.

Microorganism	Average total count
Bacteria	
(a) *Pseudomonas fluorescens *	2.4 × 10^6^ CFU/mL
(b) *Flavobacterium* sp.	
(c) *Micrococcus* sp.	
(d) *Bacillus subtilis *	

Fungi	
(a) *Aspergillus niger *	1.8 × 10^3^ colonies/mL
(b) *Aspergillus tamari *	
(c) *Aspergillus* sp.	
(d) *Penicillium* sp.	
(d) *Trichoderma* sp.	
(e) *Mucor* sp.	
